# Prevalence of inappropriate medication using Beers criteria in Japanese long-term care facilities

**DOI:** 10.1186/1471-2318-6-1

**Published:** 2006-01-11

**Authors:** Satoko Niwata, Yukari Yamada, Naoki Ikegami

**Affiliations:** 1 Department of Health Policy and Management, Keio University School of Medicine, Tokyo, Japan; 2 Department of System Management in Nursing, Graduate School of Health Sciences, Tokyo Medical and Dental University, Tokyo, Japan

## Abstract

**Background:**

The prevalence and risk factors of potentially inappropriate medication use among the elderly patients have been studied in various countries, but because of the difficulty of obtaining data on patient characteristics and medications they have not been studied in Japan.

**Methods:**

We conducted a retrospective cross-sectional study in 17 Japanese long-term care (LTC) facilities by collecting data from the comprehensive MDS assessment forms for 1669 patients aged 65 years and over who were assessed between January and July of 2002. Potentially inappropriate medications were identified on the basis of the 2003 Beers criteria.

**Results:**

The patients in the sample were similar in terms of demographic characteristics to those in the national survey. Our study revealed that 356 (21.1%) of the patients were treated with potentially inappropriate medication independent of disease or condition. The most commonly inappropriately prescribed medication was ticlopidine, which had been prescribed for 107 patients (6.3%). There were 300 (18.0%) patients treated with at least 1 inappropriate medication dependent on the disease or condition. The highest prevalence of inappropriate medication use dependent on the disease or condition was found in patients with chronic constipation. Multiple logistic regression analysis revealed psychotropic drug use (OR = 1.511), medication cost of per day (OR = 1.173), number of medications (OR = 1.140), and age (OR = 0.981) as factors related to inappropriate medication use independent of disease or condition. Neither patient characteristics nor facility characteristics emerged as predictors of inappropriate prescription.

**Conclusion:**

The prevalence and predictors of inappropriate medication use in Japanese LTC facilities were similar to those in other countries.

## Background

Inappropriate medication prescription for elderly is a major concern because it increases the risk of adverse events and health care costs [1]. Criteria defining inappropriate medication for the elderly have been developed in order to decrease its occurrence [2-5].

Beers criteria [6-8] have been most widely used to estimate prescription of potentially inappropriate medication for nursing home residents, hospital inpatients, and the community-dwelling elderly in the United States, Canada and European countries [9-47]. However, an extensive literature search did not retrieve any reports on its prevalence in Japanese long-term care (LTC) facilities which are of three types: long-term care hospitals (LTCHs), health facilities for the elderly (HFEs), and nursing homes (NHs). The care-mix among LTCHs, HFEs and NHs overlap, but LTCHs tend to care for the severer medical cases, HFEs for light care cases requiring rehabilitation, and NHs for the stable heavy care cases. There is 24 hour physician and nurse coverage in LTCHs, usually 24 hour nurse coverage but only weekday day-time physician coverage in HFEs, and only weekday work hour nurse coverage in NHs [48,49]. Regarding medications, in two of the three types of LTC facilities in Japan, LTCHs and HFEs, the cost of medication is included in the per-diem fee, so the medications prescribed are not listed on the claims forms. In the third, NHs, medication is prescribed by independent physicians and dispensed by free-standing pharmacies. Although it is theoretically possible to obtain data from the claims forms filed by the pharmacies, it has so far not been possible to link the data with the patient assessment data from the NHs. In all three types of facilities, data on diagnosis and functional status at the patient level are very difficult to obtain because there are neither uniform assessment forms nor any formal mechanisms for data collection. As a result, quality monitoring remains focused on only structural aspects, such as staffing, and there is no formal process of pharmacy reviews.

In this study, we focused on the LTC facilities that routinely use the Minimum Data Set (MDS) [50,51] as an assessment instrument for drawing care plans and for monitoring quality. The MDS includes individual patient level information, not only on health or functional status, but also on prescriptions, and has been demonstrated to be highly reliable in the Japanese population [48]. However, the number of LTC facilities that use the MDS are limited, since the form is not mandated in Japan. Therefore, the database we assembled was the only one available for evaluating the prevalence of prescription of potentially inappropriate medication for the elderly in Japanese LTC facilities and analyzing its predictors.

## Methods

### Sample

This study was conducted in 17 LTC facilities in Japan located throughout the country. We collected the MDS assessment data on 1883 patients aged 65 years and over who were assessed between January and July 2002. Because data on medication prescription for 214 patients were missing, they were excluded. As a result, the database was constructed from the data for the 1669 patients whose data were complete (477 in 8 NHs, 374 in 5 HFEs, and 818 in 4 LTCHs). There were no differences in demographic characteristics (gender, age) between the 1669 subjects of this study and the 214 who were excluded.

### Data collection

The MDS instrument provides individual level data on the following: background information, such as age, gender, payment source; patient status such as cognitive patterns, physical functioning; and the care provided. Trained staff in each facility filled in the MDS form for each patient by using information obtained through interviews, observations and chart reviews. The MDS also includes detailed information on the medication prescribed during the last 7 days, including the names and doses of the drugs prescribed, their route of administration, and total dosage. A database that included scheduled medication, non-scheduled medication, and PRN medication used at the assessment reference date was constructed. It also included oral medication, external preparations, and injections, but over-the-counter medications were excluded because the data were incomplete.

We also used the MDS ADL Self-Performance Hierarchy in the MDS assessment database, to obtain a composite score for ADL functional status [52]. The scale ranges from 0 (independent) to 6 (total dependence). In this study, a score of 2 (limited impairment) or more were classified as having an ADL disability. Cognitive impairment was assessed by the Cognitive Performance Scale (CPS) [53], which ranges from 0 (intact) to 6 (very severe impairment), and a score of 2 (mild impairment) or more were classified as cognitively impaired. Depression was scored by the Depression Rating Scale (DRS) [54], which ranges from 0 to 14, and a score of 3 or more were classified as depressed as defined by the developers of the scale.

### Measurements

We used the 3rd version of the Beers criteria [8] to identify prescription of potentially inappropriate medication, which are more useful for screening prescriptions that include potentially inappropriate medication than others. We applied the 2003 Beers criteria in this study even though the data were collected in 2002, before the publication of the 2003 version, because we concluded that the differences between the versions would have little impact in Japan since very few physicians are familiar with the Beers criteria and the later version was more comprehensive. We thought that the 2003 version served our purpose of estimating the current prevalence of inappropriate medication use in Japanese LTC facilities based on the current guidelines.

The 2003 Beers criteria consist of 2 lists. One is a list of 49 individual medications or medication classes that are inappropriate for patients 65 years or older regardless of their disease or condition. The other is a list of 56 medications or medication classes in 19 diseases or conditions for which they should be avoided.

In this study, we focused on the 30 medications or medication classes and 15 diseases or conditions. All the medications, medication classes, and diseases or conditions which were excluded from the analysis are indicated in Table 1.

**Table 1:** Inappropriate medication criteria excluded from the analysis

**Excluded from the criteria independent of disease or condition:**	
**Because the drugs were unavailable in Japan**	
Propoxyphene	Hyoscyamine
Trimethobenzamide	Tripelennamine
Carisoprodol	Dexchlorpheniramine
Medaxalone	Cyclandelate
Cyclobenzaprine	Meperidine
Chlordiazepoxide-amitriptyline	Ketorolac
Perphenazine-amitriptyline	Daily fluoxetine
Doxepine	Orphenadrine
Meprobamate	Guanethidine
Oxazepam	Guanadrel
Temazepam	Nitrofurantoin
Clidinium-chlordiazepoxide	Mesoridazine
Halazepam	Mineral oil
Chlorazepate	Amphetamines (excluding methylphenidate hydrochloride and anorexics)
**Because long-term use could not be tracked**	
Long-term use of full-dosage, longer-half life, non-COX-selective NSAIDs	
Long-term use of stimulant laxatives	
**Excluded from the criteria dependent on disease or condition:**	
**Because the drug was unavailable in Japan**	
Seizure disorder (Bupropion was unavailable.)	
**Because the patients with these disease or conditions could not be identified**	
Gastric or duodenal ulcers	SIADH/hyponatremia
Bladder outflow obstruction	

In addition, Beers criteria include medications, such as indomethacin and diphenhydramine, that are frequently used as external preparations in Japan. If limited to external use, the risk of systemic adverse effect should be low. Therefore, we decided to exclude external preparations.

### Statistical analysis

A multiple logistic regression analysis was performed to identify predictors of potentially inappropriate medication use in the patients treated with at least 1 medication. The dependent variable was inappropriate medication use independent of disease or condition. Independent variables were divided into 2 groups. The first group consisted of patient variables, such as age, gender, abnormal laboratory test results in the last 90 days (which were defined as laboratory values that were abnormal when compared to standard values), physical restraint for the last 7 days, ADL disability, cognitive impairment, depression, length of stay, number of diseases, number of medications used per day, medication cost per day, and psychotropic drug use (as defined by the Narcotics and Psychotropics Control Law in Japan). The second group consisted of facility variables and were facility type, method of reimbursement for the cost of medication, and the number of beds in the facility. Medication cost per day was converted to natural logs because it had a long-tail distribution. All variables were entered into the multiple logistic regression model by the backward stepwise method. Data were analyzed by using SPSS 12.0J software for Windows.

## Results

The demographic characteristics of the population studied are listed in Table 2. Their gender and ages were similar to data in a national survey of the same period [55].

**Table 2:** Patient characteristics

Characteristics	Number of patients as a percentage of the total (N = 1669)
Gender	
Male	25.3
Female	74.7
Age	
65 – 69	3.7
70 – 74	7.1
75 – 79	13.9
80 – 84	22.2
85 – 89	24.8
90 -	28.4
Mean age (years)	84.5
4 or more diseases	31.4
ADL score of 2 or more*	69.1
CPS score of 2 or more**	76.3
DRS score of 3 or more***	7.1
Number of medications	
1 or more	94.1
6 or more	36.2
9 or more	11.9
Psychotropic drug use	18.6

*ADL: MDS ADL Self-Performance Hierarchy

**CPS: Cognitive Performance Scale

***DRS: Depression Rating Scale

Of the 1669 patients, 356 (21.1%) were treated with at least 1 potentially inappropriate medication independent of the disease or condition in 2003 Beers criteria, and 308 (86.5%) of them treated with 1 medication on the list, 41 (11.5%) with 2, and 7 (2.0%) with 3. Table 3 shows the prevalence of prescription of each medication or medication class. The most commonly used inappropriate medication was ticlopidine, which was used by 107 patients (6.3%).

**Table 3:** Prevalence of inappropriate drug prescription (Independent of disease or condition)

Drugs	Severity*	Number of patients as a percentage of total (N = 1669)
Pentazocine	High	0.2
Muscle relaxants and antispasmodics	High	0.9
Amitriptyline	High	0.0
Long-acting benzodiazepines	High	1.9
Disopyramide	High	0.2
Digoxin >0.125 mg/d	Low	0.0
Short-acting dipyridamole	Low	0.0
Gastrointestinal antispasmodic drugs	High	0.1
Anticholinergics and antihistamines	High	1.4
Ferrous sulfate>325 mg/d	Low	0.2
Ticlopidine	High	6.3
Doxazosin	Low	1.5
Thioridazine	High	0.1
Short-acting nifedipine	High	2.0
Cimetidine	Low	1.5
Indomethacin	High	0.1
Desiccated thyroid	High	0.1
Methyldopa	High	0.1
Use of any inappropriate drugs		21.1

* Severity: Defined conceptually as a combination of both the likelihood that an adverse outcome would occur and the clinical significance of that outcome should it occur [7].

There were 300 patients (18.0%) who met the criteria for potentially inappropriate medication dependent on disease or condition. 244 (81.3%) of them were treated with 1 medication on the list, 51 (17.0%) with 2, 3 (1.0%) with 3, and 2 (0.7%) with 4. Table 4 shows the prevalence of potentially inappropriate medication use dependent on disease or condition. The highest prevalence of inappropriate medication use was for 165 among 548 patients with chronic constipation, who were prescribed inappropriate medications, such as calcium channel blockers, anticholinergics, and tricyclic antidepressants.

**Table 4:** Prevalence of inappropriate drug prescription (Dependent on disease or condition)

Disease or condition	Drug	Severity	n/N *	%
Heart failure	Disopyramide, high sodium content drugs	High	4/114	3.5
Seizures or epilepsy	Chlorpromazine, thioridazine	High	2/57	3.5
Blood clotting disorders or receiving anticoagulant therapy	Aspirin, NSAIDs, dipyridamole, ticlopidine	High	4/27	14.8
Stress incontinence**	α-Blockers, anticholinergics, tricyclic antidepressants, long-acting benzodiazepines	High	89/1028	8.7
Arrhythmias	Tricyclic antidepressants	High	1/106	0.9
Insomnia	Decongestants, theophylline, methylphenidate, MAOI, amphetamines	High	4/92	4.3
Parkinson's disease	Metoclopramide, conventional antipsychotics	High	19/88	11.4
Cognitive impairment	Barbiturates, anticholinergics, antispasmodics, muscle relaxants, CNS stimulants	High	52/1273	4.1
Depression	Sympatholytic agents	High	8/119	6.7
Syncope or falls	Short to intermediate-acting benzodiazepine, tricyclic antidepressants	High	23/103	22.3
COPD	Long-acting benzodiazepines, β-blockers	High	1/44	2.3
Chronic constipation	Calcium channel blockers, anticholinergics, tricyclic antidepressants	Low	165/548	30.1
Use of any of above for each disease or condition			300/1669	18.0

* N: Number of patients with the disease or condition. n: Number of patients treated with any of drug(s) that are inappropriate for that disease or condition.

** We could not identify the patients with 'stress incontinence' because the MDS data were limited to 'incontinence' in general.

Table 5 shows the results of the multiple logistic regression analysis to identify predictors of inappropriate medication use, independent of disease or condition. Psychotropic drug use (OR = 1.511), medication cost per day (OR = 1.173) and the number of medications used per day (OR = 1.140) increased the risk of inappropriate medication use, while age (OR = 0.981) decreased the risk.

**Table 5:** Multiple logistic regression analysis to identify predictors of inappropriate drug use

Variable	Odds ratio	95%CI	P value	Correlation coefficient*
Psychotropic drug use	1.528	1.133 – 2.059	0.005	0.156
Medication cost per day**	1.172	1.011 – 1.360	0.036	0.213
Number of medications per day	1.141	1.075 – 1.211	0.000	0.268
Age	0.981	0.965 – 0.998	0.029	-0.124

* The Pearson's bivariate correlation coefficients.

** Cost of medication per day has been converted to natural logs.

## Discussion

### Prevalence of potentially inappropriate medication use independent of disease or condition

The prevalence of prescription of potentially inappropriate medication based on the 2003 Beers criteria were 13.4% in the United States [46], and 5.8 to 25.7% in 8 European countries [42]. On earlier versions of the criteria, they were 10.5 to 54.7% in patients in nursing homes [9,14,21,26,27,29,30,33,39] and 2.2 to 35.6% in patients in the community [10-12,16-18,20-25,28,31,32,34-37,40-46]. The prevalence in this study was essentially the same. Parenthetically, there were 5 (0.2%) terminally ill patients in the sample but inappropriate medication was not prescribed for this group.

The most commonly inappropriately prescribed medication was ticlopidine. By contrast, ticlopidine is rarely used in the United States, because clopidogrel, a safer alternative to aspirin, is available. However, clopidogrel was not available in Japan at the time of the study, which may have led to a higher prevalence of inappropriate use than would have been the case if it had been available. The fact that ticlopidine was also commonly prescribed in Italy [30,42], where clopidogrel was also unavailable, may provide support for this hypothesis. When ticlopidine was excluded from the list, the prevalence of potentially inappropriate medication use independent of the disease or condition decreased from 21.1% to 16.4%, thus remaining in the range of previous studies.

Anticholinergics and antihistamines, long-acting benzodiazepines, short-acting dipyridamole, and short-acting nifedipine were other medications on the list that were frequently used. The prevalence of inappropriate prescription of dipyridamole and nifedipine use in this study was slightly higher than in other studies. Propoxyphene was commonly used in the United States, but was not prescribed for the subjects of this study because it was unavailable in Japan. The prevalence of the antiarrhythmic agent, amiodarone, added to the 2003 Beers criteria, was also high in previous studies [42,46], but none of the patient were treated with it in our study because the national formulary regulation in Japan restricts its use to cases in which other medications have proved ineffective.

### Factors associated with inappropriate medication use independent of the disease or condition

The result of the multiple logistic regression analysis in this study identified psychotropic drug use, number of medications per day, medication cost per day, and age as factors associated with inappropriate medication prescription in LTC facilities, which was the same as in other countries [9,10,15,18,19,21,25-32,35-39,42,43,46]. When ticlopidine was excluded from the analysis, the results did not change greatly, but age and cost of medication per-day were excluded.

It should be noted that the other patient variables and the 3 facility variables were not selected in the multiple logistic regression analysis. Since Japanese LTC facilities differ not only in professional staffing levels and medical need, but in reimbursement for medication costs, we expected these characteristics to be reflected in the prescription pattern. The fact that these variables were not included indicates that the prescription pattern in Japanese LTC facilities depends on other factors, such as the prescribing habits of individual physicians.

### Limitations

The first limitation of this study is that the sample facilities in this study may have been of higher quality, both in the standard of care and prescribing habits, which would lead to a lower prevalence of inappropriate medication prescription. Second, many have noted that the Beers criteria do not include drug-drug interactions or underprescribing [10,11,27,29,30,35,40,46]. Third, there may be racial differences in some drugs metabolizing enzymes [56,57] that affect the incidence of adverse effects as well as the dosage limitations on the list. Finally, there may be potentially inappropriate medications for elderly that are available in Japan but not in the United States, and if there are, modifications of the Beers criteria based on expert opinion would be needed for application to Japan. However, the Japan Geriatrics Society had just published guidelines for appropriate medication prescription for the elderly when this paper was submitted [58]. They are a modification of the 2003 Beers criteria, and the majority of the guidelines appear to follow the original criteria with some changes in dose and the addition of several medications. Thus, although we used the original Beers criteria to make our study comparable to international studies, our results appear to be generally applicable to the Japanese situation.

## Conclusion

By focusing on LTC facilities that used the MDS comprehensive assessment form, we were able to confirm for the first time that the prevalence of inappropriate medication prescription in Japanese LTC facilities was 21% according to the criteria independent of disease or condition and 18% according to the criteria dependent on disease or condition, based on the 2003 Beers criteria. The results of a multiple logistic regression analysis indicated that psychotropic drug use and the number of medications prescribed per day are risk factors of potentially inappropriate medication use independent of disease or condition. These results are similar to those of previous studies in other countries.

## Competing interests

The author(s) declare that they have no competing interests.

## Authors' contributions

SN constructed the database, performed the statistical analysis, and drafted the manuscript. YY assisted in performing the analysis and drafting the manuscript. NI conceived the study design, obtained the data, and assisted in drafting the manuscript. All authors read and approved the final manuscript.

## Pre-publication history

The pre-publication history for this paper can be accessed here:


